# Impact of treatment on progression to castration‐resistance, metastases, and death in men with localized high‐grade prostate cancer

**DOI:** 10.1002/cam4.981

**Published:** 2016-12-20

**Authors:** Eric T. Miller, Karim Chamie, Lorna Kwan, Michael S. Lewis, Beatrice S. Knudsen, Isla P. Garraway

**Affiliations:** ^1^ Department of UrologyDavid Geffen School of Medicine at UCLALos AngelesCalifornia; ^2^ Department of PathologyGreater Los Angeles Veterans Affairs Health SystemLos AngelesCalifornia; ^3^ Department of Pathology and Laboratory MedicineCedars‐Sinai Medical CenterLos AngelesCalifornia; ^4^ Jonsson Comprehensive Cancer CenterDavid Geffen School of Medicine at UCLALos AngelesCalifornia; ^5^ Division of UrologyGreater Los Angeles Veterans Affairs Healthcare CenterLos AngelesCalifornia

**Keywords:** ADT, disease progression, HGPC, risk

## Abstract

Men with high‐grade prostate cancer (HGPC) are at greatest risk of disease progression. Clinical risk factors associated with castration‐resistant prostate cancer (CRPC), metastases, and prostate cancer‐specific mortality (PCSM) were identified in a contemporary HGPC cohort. Clinical data was collected from men diagnosed with Gleason sum (GS) ≥8 at the Greater Los Angeles Veterans Affairs (GLA‐VA) Healthcare System between 2000 and 2013. Multivariable competing risks regression analyses assessed progression to CRPC, metastases, and PCSM within three treatment strata. The cumulative incidence of disease progression was calculated at 2, 5, and 10‐year time points. Review of 2149 prostate cancer cases yielded 322 with HGPC. Median survival times for cancer‐specific and overall mortality were significantly shorter in men treated with primary androgen deprivation therapy (ADT) (*P* = 0.0002 and *P* < 0.0001). Multivariable analyses revealed that clinical stage N1, GS 10, and treatment with primary ADT were significantly associated with increased risk of CRPC, metastases, and PCSM. Significant differences in these outcomes were not observed in men treated with radical prostatectomy (RP) when compared to those treated with radiation therapy combined with short‐term ADT (XRT‐ADT). Ten‐year event rates of progression to CRPC, metastases, and PCSM, for men treated with primary ADT, were 45.5%, 25.4%, and 25.1%, respectively. In conclusion, GS 10 and lymph node involvement, as well as primary ADT treatment in men with HGPC was associated with increased risk of CRPC, metastases, and PCSM. Curative‐intent treatment with RP or XRT‐ADT is associated with reduced progression rates and death in men with HGPC.

## Introduction

To predict the risk of disease recurrence following definitive treatment for localized prostate cancer (PC), risk stratification tools often integrate Gleason sum (GS), prostate specific antigen (PSA), and clinical tumor (cT) stage 1, 2, 3. In the commonly used D'Amico risk classification, these variables enable newly diagnosed men to be assigned to low (GS <6, PSA<10 ng/mL, cT1c or cT2a), intermediate (GS 7, PSA 10‐20 ng/mL, cT2b), or high (GS ≥8, PSA ≥20 ng/mL, ≥cT2c) risk groups following treatment 3. In the current PSA screening era, approximately 15% of men with newly diagnosed PC display high‐risk disease 4, 5. Since metastatic progression is often observed within 5 years of treatment, it is postulated that many who fail curative‐intent therapy harbor micro‐metastatic disease at diagnosis that is undetectable with standard‐of‐care imaging modalities 6. In order to improve disease control in such ‘very’ high‐risk cases, combinational approaches will be needed 7, 8.

Most risk stratification schemes place men at high risk of progression if high‐grade PC (HGPC), designated by pathological Gleason sum ≥8, is identified in diagnostic biopsy and/or prostatectomy specimens 1, 2, 3, 9. However, HGPC is a heterogeneous disease, representing a wide range of pathological features and clinical outcomes 10, 11. Intervention with curative‐intent therapies, including radical prostatectomy (RP) or external beam radiation therapy combined with short‐term androgen deprivation therapy (XRT‐ADT), is associated with 10‐year PC survival rates as high as 98%, despite biochemical recurrence (BCR) in approximately one‐third of these men. Thus, sequential rises in PSA following treatment for PC, which defines BCR, are not always associated with metastatic progression or shorter survival 6, 9. Notably, metastasis‐free survival intervals of 10 years have been observed in men with BCR following RP, even in the absence of salvage therapies (XRT and/or ADT) 12, 13. Recent results from the STAMPEDE clinical trial have demonstrated that the addition of docetaxel to standard treatments for high‐risk, non metastatic PC is associated with a significantly improved failure‐free survival 14. If the results of this randomized‐controlled trial are further validated, there may be a paradigm shift in the clinical management of high‐risk patients. However, given that only a subset of men with BCR experience lethal progression, improved tools are needed to delineate men at highest risk of castration‐resistant PC (CRPC), metastases, or death from PC. These tools will help to target men most likely to benefit from combinatorial treatment approaches and prevent unnecessary exposure to adverse effects associated with adjuvant and/or salvage therapies 4, 7, 14, 15.

In the current study, we examined a diverse cohort of men with localized HGPC who were diagnosed and treated within the Greater Los Angeles Veterans Affairs (GLA‐VA) Healthcare System between 2000 and 2013. All cases are part of a biorepository that was designed to facilitate future molecular profiling of PC specimens. Disease progression outcomes were analyzed by three treatment strata: RP, XRT‐ADT, and primary androgen deprivation therapy (ADT). Given the heterogeneous nature of HGPC, we hypothesized that clinical variables present at diagnosis, as well as treatment choice, a variable that is not commonly assessed in PC outcomes studies but closely linked with metastases and prostate cancer‐specific mortality (PCSM), are associated with time to CRPC. The overall goal of this study was to identify variables associated with meaningful clinical progression (CRPC, metastases, death from PC) in men diagnosed with HGPC.

## Methods

### Study population

**Figure 1:**
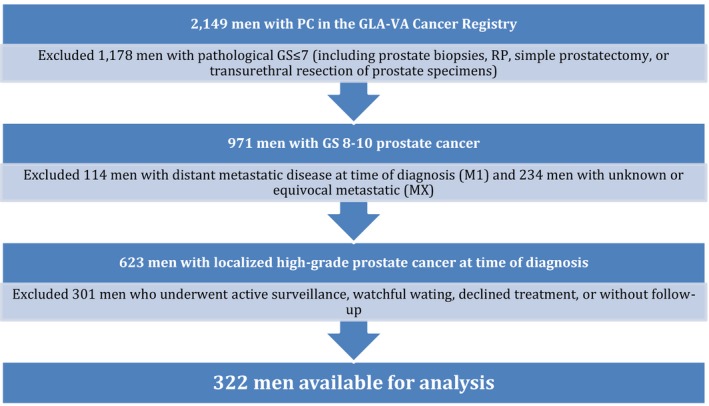
High Grade Prostate Cancer Cohort Selection.

After obtaining institutional review board approval at the GLA‐VA, the Cancer Registry, housed at the GLA‐VA, was queried to identify men diagnosed with PC between 2000 and 2013 (*n *=* *2,149). Electronic medical record review enabled abstraction of demographic and clinical variables and generation of a GLA‐VA PC database. We excluded cases with distant metastatic disease at diagnosis (clinical stage M1, *n *=* *114), cases for which metastatic burden was not assessed or demonstrated equivocal results (clinical stage Mx, *n *=* *234), and those who received active surveillance, watchful waiting, declined treatment, or left the VA healthcare system (*n *=* *301). To derive the cohort with HGPC used for this study, cases with diagnostic pathological reports documenting a GS≤7 (*n *=* *1,178) were excluded. Only cases with Gleason sum ≥8 (denoted on the pathological reports from prostate biopsies, RP, simple prostatectomy, or transurethral resection of prostate specimens) were included for the final study population, which resulted in 322 men (Fig. 1).

### Variables

#### Race/ethnicity

Race and ethnicity were abstracted from the VA Computerized Patient Record System (CPRS), where race was recorded as White, Black or African American, Asian, Native Hawaiian or other Pacific Islander, Native American, Unknown, or Unanswered. Ethnicity was recorded as Hispanic or Latino, not Hispanic or Latino, unknown, or unanswered. For the HGPC cohort, race was categorized as White, Black, and Other.

#### Clinical Stage and Tumor Characteristics

The clinical tumor (cT) stage was dichotomized into T1/T2 and T3/T4. PSA at the time of diagnosis was dichotomized into ≤20 ng/mL and >20 ng/mL. Gleason sum was categorized as 8, 9, or 10. The centralized pathological review was performed on a subset of cases (approximately 20%) by a genitourinary pathologist who was blinded to clinical outcomes in order to confirm appropriate and consistent designation of pathological grade. To assign the clinical lymph node (N) stage, diagnostic computed tomography (CT) or magnetic resonance imaging (MRI) scans were reviewed. If lymph node metastases were not identified, the case was designated as N0. If pelvic lymph node metastases were present, the case was designated as N1. If imaging studies were absent or inconclusive, the cases were designated as Nx. Pathological N‐stage, obtained from patients who underwent RP with concurrent pelvic lymph node dissection, was not considered in this analysis.

#### Type of primary treatment

The primary definitive treatment following a diagnosis of PC was abstracted from CPRS. Men were categorized by primary treatment received, including RP, XRT‐ADT, and primary continuous ADT. Men treated with XRT‐ADT received the standard of care external beam radiation and short‐term ADT at the discretion of the treating physicians.

#### Disease progression

The development of CRPC and metastases were documented as yes/no events. The development of CRPC was defined as (1) two sequential rises in PSA, (2) metastatic progression while on ADT, or (3) initiation of CRPC treatment, whichever came first. Metastatic disease was confirmed by sequential imaging modalities (i.e., Technetium‐99 bone scan, CT or MRI scans). Length of follow‐up was calculated for each outcome from date of diagnosis to date of disease progression, or to date of most recent clinical contact for those without progression.

#### Mortality

Each chart was independently reviewed by the primary and senior author to assess specific cause of death and coded as follows: alive, death from PC, or death from other cause. Death from PC was noted if the patient was documented to have metastatic disease and died in hospital of related complications or died in hospice care. Survival time was computed from date of diagnosis to the date of death, or from date of diagnosis to the date of most recent clinical contact for censored cases.

### Statistical analysis

The HGPC cohort was separated into three subcohorts based upon treatment received: RP, *n *=* *106 (33%); XRT‐ADT, *n *=* *138 (43%); primary ADT, *n *=* *78 (24%). Bivariable analysis compared patient characteristics across the sub‐cohorts, using chi‐square and Fisher's exact tests for categorical variables and ANOVA for continuous variables. Kaplan–Meir survival analysis was performed for PCSM and all‐cause mortality stratified by treatment received. Multivariable competing risks regression models were constructed to identify variables associated with the development of CRPC, metastases, and PCSM. Death from other causes was coded as the competing risk for disease progression analyses and non‐PC mortality was coded as the competing risk in PCSM analysis. Variables evaluated in the models included age at diagnosis, race (White as referent), dichotomized PSA (≤20 ng/mL as referent), Gleason sum (eight as referent), dichotomized T‐Stage (T1/T2 as referent), clinical N‐Stage (N0 as referent), and primary treatment received (XRT‐ADT as referent). Cumulative incidence curves for each subcohort were generated from the resulting models. Predicted 2, 5, and 10‐year progression event rates were calculated for CRPC, metastases, and PCSM for each treatment strata. All tests were two‐sided and a statistical *P *< 0.05 was used to denote significance. Analyses were performed, using Stata 13.1 (StataCorp LP, College Station, TX) and SAS 9.4 (SAS Institute Inc., Cary, NC).

## Results

### Patient characteristics

Clinical and pathological characteristics from the study subjects stratified by the three treatments received are shown in Table 1. The median age of the HGPC cohort was 66.2 years (range 38.4–90.4 years). Men treated with primary ADT were more likely to be older, Black, and have clinical N1 stage compared to men treated with XRT‐ADT and RP (*P* < 0.0001, *P* = 0.0393 and *P* = 0.0047, respectively). There were no statistical differences in Gleason sum, time to CRPC, time to metastases, time to death from PC, or time to death from any cause among the treatment groups. Overall, 15.3% of men in the HGPC cohort developed CRPC, 14.2% developed metastases, and 9.7% experienced PCSM.

**Table 1:** Patient characteristics stratified by primary treatment received

Initial Stage and Treatment	Total	RP	XRT‐ADT	ADT	*P‐value*
	*N* = 322 (100%)	*N* = 106 (33%)	*N* = 138 (43%)	*N* = 78 (24%)	
Age					<0.0001
Mean (SD)	67.3 (8.4)	62.8 (6.7)	68.3 (7.4)	71.7 (9.4)	
Median	66.2	63.7	67.6	72.3	
Range	38.0–90.4	38.0–78.2	49.0–84.3	51.3–90.4	
Race					0.0393
White	151 (46.9%)	55 (51.9%)	70 (50.7%)	26 (33.3%)	
Black	133 (41.3%)	40 (37.7%)	56 (40.6%)	37 (47.4%)	
Other	38 (11.8%)	11 (10.4%)	12 (8.7%)	15 (19.2%)	
Clinical T‐Stage					0.0304
T1 or T2	273 (86.4%)	95 (91.4%)	118 (87.4%)	60 (77.9%)	
T3 or T4	43 (13.6%)	9 (8.7%)	17 (12.6%)	17 (22.1%)	
Clinical N‐Stage					0.0047
N0	213 (66.2%)	74 (69.8%)	101 (73.2%)	38 (48.7%)	
N1	54 (16.8%)	16 (15.1%)	20 (14.5%)	18 (23.1%)	
Nx	55 (17.1%)	16 (15.1%)	17 (12.3%)	22 (28.2%)	
Gleason sum					0.3962
8	195 (60.6%)	66 (62.3%)	83 (60.1%)	46 (59.0%)	
9	120 (37.3%)	40 (37.7%)	50 (36.2%)	30 (38.5%)	
10	7 (2.2%)	0 (0%)	5 (3.6%)	2 (2.6%)	
PSA Category (ng/mL)					<0.0001
≤20	244 (76.5%)	98 (95.2%)	107 (77.5%)	39 (50.0%)	
>20	75 (23.5%)	5 (4.9%)	31 (22.5%)	39 (50.0%)	
PSA at Diagnosis (ng/mL)					<0.0001
Median	10.0	6.7	9.8	19.9	
Range	0.1–1329.0	1.7–53.3	0.1–172.4	3.8–1328.0	
Development of CRPC					<0.0001
No	261 (84.7%)	95 (93.1%)	119 (88.2%)	47 (66.2%)	
Yes	47 (15.3%)	7 (6.9%)	16 (11.9%)	24 (33.8%)	
Time to CRPC (mo)					0.8112
Mean (SD)	38.3 (30.6)	46.0 (40.1)	37.5 (25.3)	37.0 (32.4)	
Median	32.0	32.3	30.7	32.7	
Range	3.7–128.2	13.3–119.1	7.2–90.7	3.7–128.2	
Development of Metastases					0.0052
No	273 (85.8%)	92 (87.6%)	125 (90.6%)	56 (74.7%)	
Yes	45 (14.2%)	13 (12.4%)	13 (9.4%)	19 (25.3%)	
Time to Metastases (mo)					0.9600
Mean (SD)	52.0 (42.8)	54.3 (52.6)	49.1 (33.6)	52.4 (43.0)	
Median	39.6	40.4	37.3	39.9	
Range	2.3–174.6	4.6–174.6	16.4–106.5	2.3–160.5	
Vital Status					<0.0001
Alive	214 (66.9%)	93 (87.7%)	89 (64.5%)	32 (42.1%)	
Death from PC	31 (9.7%)	5 (1.6%)	10 (7.3%)	16 (21.1%)	
Death from any cause	75 (23.4%)	8 (7.6%)	39 (28.3%)	28 (36.8%)	
Time to Death from PC (mo)					0.7274
Mean (SD)	63.7 (40.4)	77.1 (46.8)	62.3 (34.1)	60.3 (43.8)	
Median	56.2	48.7	59.9	53.4	
Range	12.1–165.2	39.2–143.5	19.0–122.8	12.1–165.1	
Time to Death from any cause (mo)					0.1291
Mean (SD)	59.9 (38.6)	65.8 (36.5)	66.4 (38.0)	50.9 (38.9)	
Median	51.6	54.1	60.4	43.1	
Range	2.4–170.0	12.7–143.5	9.3–170.0	2.4–165.2	
Length of Follow‐up (mo)					0.0103
Mean (SD)	67.1 (39.2)	63.6 (35.7)	74.4 (39.7)	58.8 (41.0)	
Median	58.2	52.4	64.6	51.6	
Range	0.0–185.0	1.3–174.6	0.0–185.0	2.4–173.4	

ADT, primary androgen deprivation therapy; RP, radical prostatectomy; XRT‐ADT, radiation therapy combined with short‐term ADT; CRPC, castration‐resistant prostate cancer; PC, prostate cancer; mo, months; PSA, prostate specific antigen

### Disease progression and survival analysis

**Figure 2:**
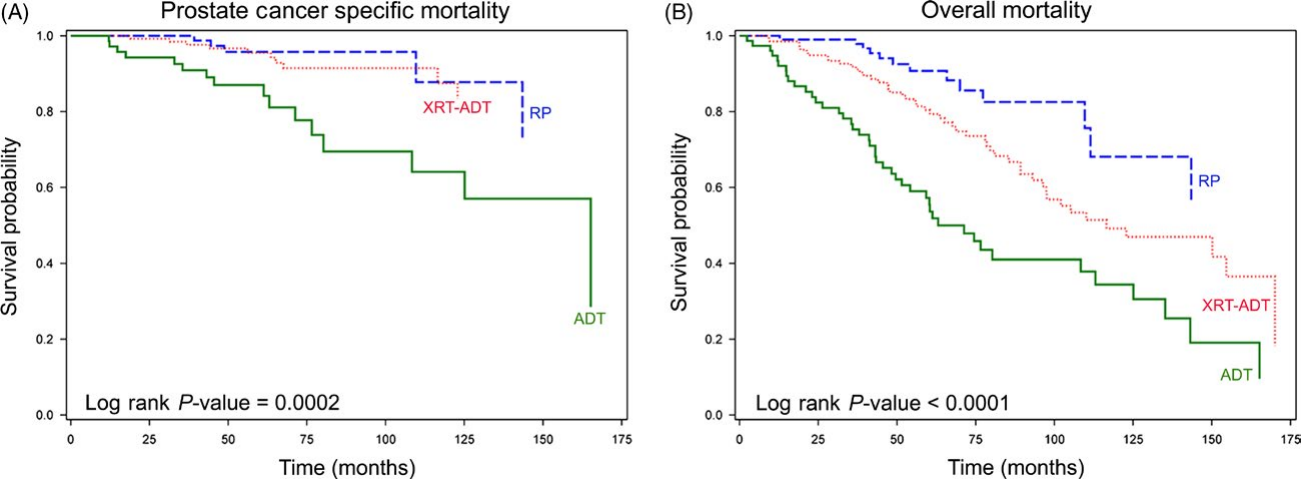
Kaplan‐Meier survival analysis stratified by treatment received. (A) Greater than 50% of patients treated with RP and XRT‐ADT were alive at time of follow‐up. Patients treated with primary ADT were significantly more likely to experience prostate cancer‐specific mortality(PCSM) (*P* = 0.0002) with a median survival of 165 months. (B) Median overall survival was not reached in men treated with RP. Median survival was ~116 months in men who received treatment with XRT‐ADT. Men treated with primary ADT had significantly worse overall survival of 71 months (*P* < 0.0001). ADT, primary androgen deprivation therapy; RP, radical prostatectomy; XRT‐ADT, radiation therapy combined with short‐term ADT.

Kaplan–Meier survival analysis for PCSM stratified by treatment received revealed that median survival was never reached in the RP and XRT‐ADT treatment groups by the end of follow‐up period (i.e. >50% of the patients were still alive) (Fig. 2). However, the median time to PCSM was 165 months for men who received primary ADT, which was significantly shorter than for men treated with RP and XRT‐ADT (*P* = 0.0002). The median time to all‐cause mortality was 116 months for men who received XRT‐ADT and 71 months for those who received primary ADT (*P* < 0.0001). Approximately 57% of the RP subcohort did not die during the course of the follow‐up period.

**Figure 3:**
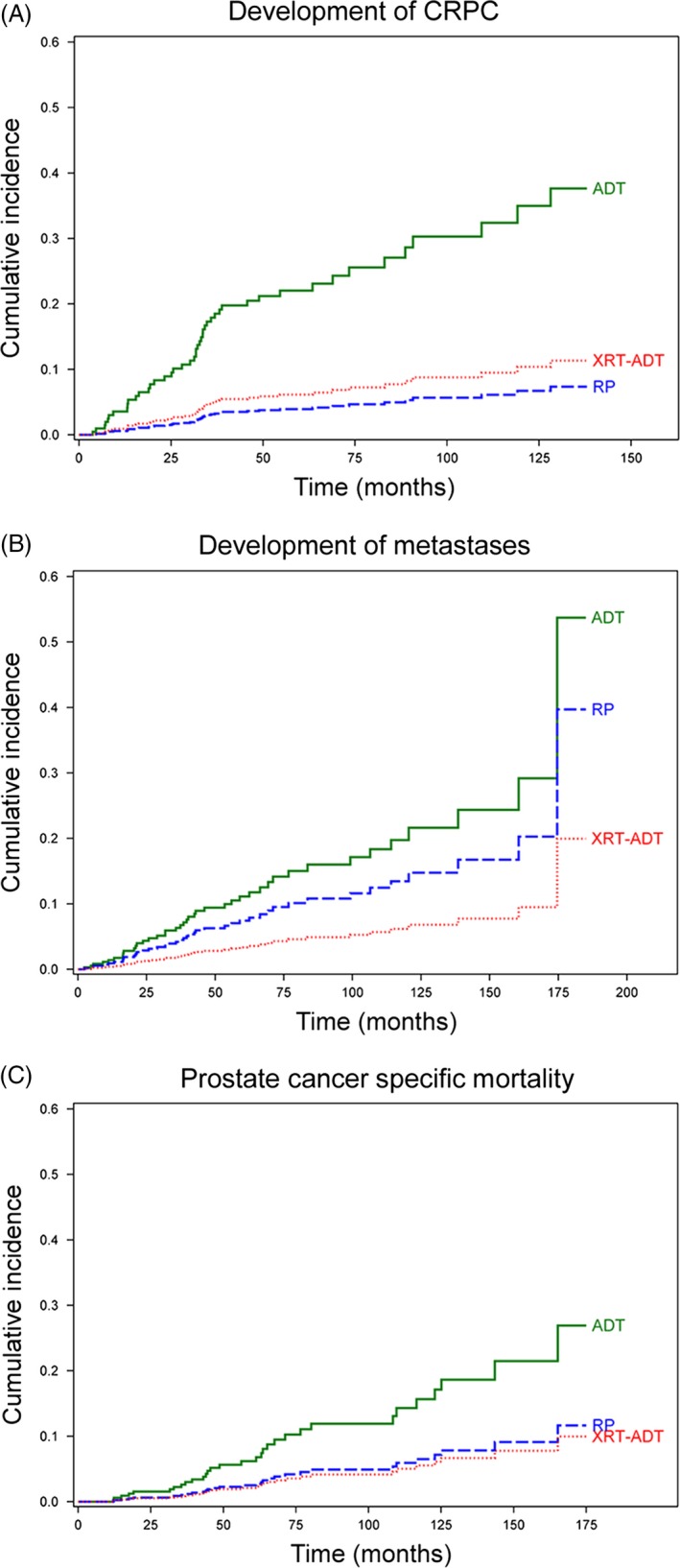
Competing Risks‐Regression Analysis Stratified by Treatment Received. (A) Treatment with primary ADT was significantly associated with more rapid progression to CRPC (HR 3.92, CI 95% 1.80–8.54), while treatment with RP or XRT‐ADT was not. (B) Treatment with primary ADT was significantly associated with more rapid progression to metastases (HR 3.46, CI 95% 1.38–8.69), while treatment with RP or XRT‐ADT was not. (C) Treatment with primary ADT was significantly associated with more rapid progression to prostate cancer‐specific mortality(PCSM) (HR 2.98, CI 95% 1.15–7.73), while treatment with RP or XRT‐ADT was not. ADT, androgen deprivation therapy; RP, radical prostatectomy; XRT‐ADT, radiation therapy combined with short‐term ADT; CRPC, castration‐resistant prostate cancer.

Results from the multivariable competing risks regression analyses are shown in Table 2. There was an increased risk for all progression events in men with N1 and GS 10 disease. Cumulative incidence curves from the competing risks regression analyses and predicted 2, 5, and 10‐year disease event progression rates for CRPC, metastases and PCSM stratified by treatment received are shown in Figure 3 and Table 3, respectively.

**Table 2:** Multivariable competing‐risks regression analysis

Variables	CRPC	Metastases	PSCM
	HR (95% CI)	HR (95% CI)	HR (95% CI)
Age	0.98 (0.94–1.02)	0.97 (0.93–1.01)	0.98 (0.93–1.02)
Race			
White	–	–	–
Black	0.85 (0.46–1.56)	0.62 (0.33–1.17)	0.73 (0.35–1.54)
Other	0.36 (0.10–1.31)	0.25 (0.06–1.13)	0.32 (0.06–1.67)
Clinical T Stage			
T1 or T2	–	–	–
T3 or T4	1.30 (0.51–3.35)	1.11 (0.39–3.19)	1.03 (0.32–3.27)
Clinical N‐Stage			
N0	–	–	–
N1	2.35 (1.13–4.88)	2.80 (1.26–6.24)	3.09 (1.19–8.06)
Nx	0.81 (0.33–1.99)	1.84 (0.79–4.29)	1.37 (0.48–3.93)
Gleason Sum			
8	–	–	–
9	1.60 (0.81–3.19)	1.18 (0.55–2.53)	1.37 (0.55–3.41)
10	4.32 (1.07–17.45)	8.82 (2.16–36.04)	6.63 (1.43–30.66)
PSA Category (ng/mL)			
≤20	–	–	–
>20	1.37 (0.61–3.08)	1.74 (0.72–4.24)	1.83 (0.64–5.24)
Primary Treatment			
XRT‐ADT	–	–	–
RP	0.63 (0.23–1.71)	2.27 (0.94–5.47)	1.18 (0.37–3.80)
ADT	3.92 (1.80–8.54)	3.46 (1.38–8.69)	2.98 (1.15–7.73)

ADT, primary androgen deprivation therapy; RP, radical prostatectomy; XRT‐ADT, radiation therapy combined with short‐term ADT; CRPC, castration‐resistant prostate cancer; PC, prostate cancer; mo, months; PSA, prostate specific antigen; PCSM, prostate cancer specific mortality.

**Table 3:** Multivariable predicted disease progression event rates stratified by treatment received

	CRPC			Metastases			PCSM		
	2‐year	5‐year	10‐year	2‐year	5‐year	10‐year	2‐year	5‐year	10‐year
RP	1.8%	4.8%	8.1%	3.5%	8.9%	17.5%	1.0%	3.1%	9.0%
XRT‐ADT	2.8%	7.5%	12.6%	1.5%	4.0%	8.1%	0.9%	2.8%	8.2%
ADT	12.1%	29.7%	45.5%	5.2%	13.2%	25.4%	3.1%	9.1%	25.1%

ADT, primary androgen deprivation therapy; RP, radical prostatectomy; XRT‐ADT, radiation therapy combined with short‐term ADT; CRPC, castration‐resistant prostate cancer; PCSM, prostate cancer specific mortality.

## Discussion

Men with newly diagnosed localized HGPC may select management of their disease with curative intent treatments such as RP, XRT‐ADT, or opt for observation, surveillance, and/or palliation. It is well documented that men with HGPC at the time of biopsy or RP have a high risk of BCR and clinical progression 18. However, it is important to consider that not all of these patients will, in fact, recur; and for those with BCR, not all will experience metastatic or lethal progression 10. New data from the STAMPEDE clinical trial have demonstrated a significant reduction in disease recurrence in men with high‐risk, localized PC treated with upfront docetaxel in combination with standard of care therapy 14. Since high‐risk disease (which includes men with HGPC) accounts for approximately 15% of new localized PC diagnoses, it is important to attempt sub‐stratification of these men in order to identify those most likely to benefit from upfront combinatorial therapy and reduce morbidity associated with adverse effects from chemotherapy or other adjuvant treatments 19, 20, 21.

In accordance with previous studies, we found few clinical or pathological variables associated with progression to CRPC, metastases or death from PC in the analysis of this HGPC cohort 9. However, multivariable analysis controlling for treatment received revealed a significant association with GS 10 tumors and poorer outcomes. The prognostic value of Gleason score remains a principal reason why it is integrated into risk stratification 2, 3, 7, 22, 23. While studies consistently demonstrate that GS 8–10 tumors are associated with worse prognosis, there remains a wide distribution of outcomes among men with HGPC 11, 24. This was observed in our analysis, where GS 10 was the only pathological feature associated with disease progression. This finding demonstrates that the highest Gleason score along the continuum correlates with a higher risk of progression. However, since men who progressed also displayed GS 8 and GS 9 tumors, aspects of tumor biology that are not captured with the Gleason score alone may contribute to outcome as well.

Previous analysis of VA men with Gleason sum ≥8 disease identified in the SEARCH database that were treated with RP revealed that adverse pathological features found in the surgical specimen, including seminal vesicle invasion, extracapsular extension, or positive surgical margins, were associated with increased risk of early BCR 10. Although these features are useful for identifying RP patients that may benefit from combinatorial therapies, they cannot be used for men who chose nonsurgical PC management. Additional factors that can be determined at diagnosis are needed to predict prognosis and assist with management decisions in nonsurgical HGPC cases, especially if new clinical trial evidence will encourage a more aggressive approach for those with newly diagnosed HGPC.

Our results demonstrated that men treated with primary ADT had worse outcomes, including a higher rate of CRPC, metastasis, and death from PC. These findings suggest that RP or XRT‐ADT may be superior treatment choices for men with HGPC. The role of primary ADT in nonmetastatic PC is controversial without clear evidence of offering a survival benefit, however, some men are offered and accept this choice of treatment for different reasons, such as suspicion of micrometastatic disease due to high PSA at diagnosis, or patient preference for a noninvasive treatment option 16, 17. ADT is often associated with multiple adverse effects, including increased risk of cardiovascular disease, venous thromboembolism, peripheral arterial disease, and negative quality of life factors 25, 26, 27. Therefore, it is important to consider possible adverse effects of ADT and the increased rate of lethal progression when managing localized HGPC.

Several studies have evaluated the benefit of curative‐intent therapy in men with high‐risk PC. In randomized trials based in Scandinavia, the UK, and North America, there were significant improvements in disease‐free and overall survival in men treated with combination radiation therapy and ADT compared with ADT alone 28, 29. Additionally, several large retrospective studies demonstrated improved survival rates in high‐risk men treated with RP, with 10‐year cancer‐specific survival ranging from 82% to 92% 19, 30, 31. The results of our retrospective analysis are consistent with these reports, and our survival outcomes among men treated with curative‐intent therapy were comparable.

A noteworthy finding among this diverse cohort of men was the lack of association between race and disease progression. Our study revealed equal distribution of HGPC among Black and White men and race was not a factor that contributed to risk of CRPC, metastasis, or PCSM 32, 33, 34. These results are consistent with a prior study of men with nonmetastatic PC diagnosed and treated within the GLA‐VA Healthcare System, which also found no racial differences associated with HGPC and outcome 35. However, closer evaluation of the distribution of GS among all men diagnosed and treated with PC at GLA‐VA between 2000 and 2013 is needed to make firm conclusions that similar proportions of White and Black men display HGPC. Our analysis did, in fact, reveal differences in treatment selection associated with race. Significantly fewer Black men in the HGPC cohort were treated with RP, opting for XRT‐ADT or primary ADT. Identifying reasons for this observation are beyond the scope of this study, however previous analyses indicate that they are likely multifactorial 36, 37. Dedicated studies focused on close examination of PC racial disparities within the VA are needed in order to better understand a potential impact on tumor biology and/or clinical outcome.

Our retrospective outcomes analysis has some specific limitations. The HGPC cohort includes a wide range of patients diagnosed and treated by many different healthcare providers within the GLA‐VA, so there may be inconsistencies in designation of clinical T‐stage based upon clinical examination. Although all patients in our study underwent metastatic evaluation, including documented bone, CT and/or MRI scans at the time of diagnosis, there was no standardized protocol for monitoring patients following treatment. Follow‐up PSA testing and metastatic imaging were performed at the discretion of the treating physician, so the precise timing of disease recurrence and/or progression may not be accurately reflected in the dates of PSA or imaging results. Other confounding factors that were not included in this study were comorbidities, performance status, and socioeconomic status. Finally, the fact that our cohort was composed entirely of Veterans may raise concern about the generalizability of our results to other populations of men with HGPC. However, the single institution also represents the strength of this study because it enables maximal length of follow‐up, and the options for care and delivery are relatively consistent. By conducting competing risks analyses, we have accounted for alternative causes of death (other than PC), which strengthens the validity of our results. Thus, comparison across treatment sub‐cohorts in this VA study is relevant and comparable with other VA and non‐VA studies focused on outcomes of men with high risk PC.

In conclusion, clinical stage N1, GS 10, and treatment with primary ADT were associated with lethal PC progression in a contemporary cohort of men diagnosed and treated for HGPC in an equal access VA healthcare system. Men with HGPC who receive definitive therapy (RP or XRT‐ADT) had significantly improved PC‐specific and overall survival compared to men treated with primary ADT. These findings suggest that curative‐intent treatments should be considered in men with newly diagnosed HGPC. However, a subset of men with HGPC will progress to CRPC, metastases, or death from PC despite receiving curative‐intent treatment. This subset will benefit from improved risk stratification and potential combinatorial therapies. Given the limited contribution of clinical variables in predicting lethal progression in HGPC cases, future integration of molecular profiles generated from PC specimens linked to this dataset may help generate improved classifiers for men with newly diagnosed HGPC and designate men at highest risk of disease progression.

## Conflicts of Interest

None.
